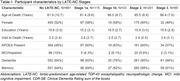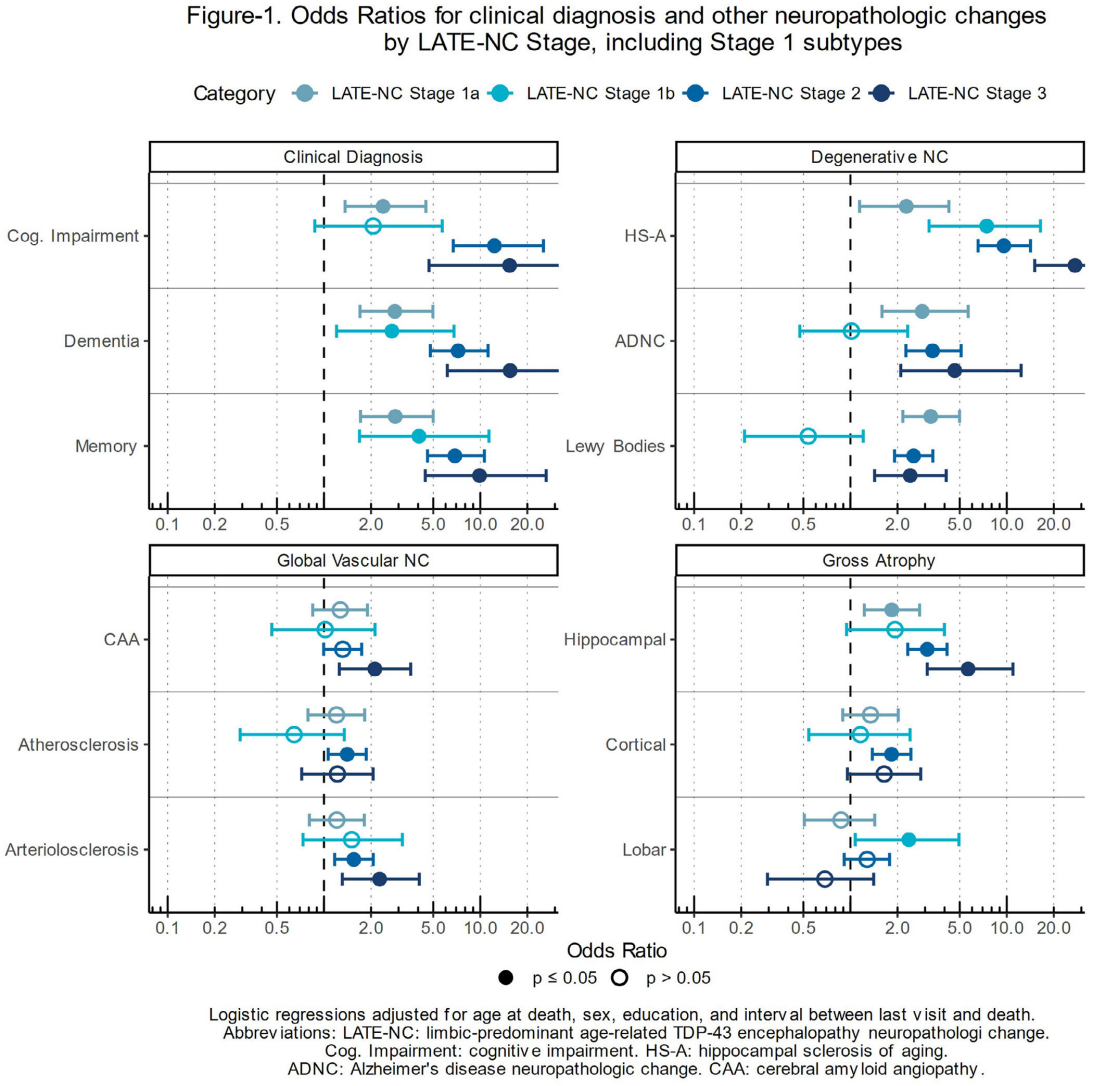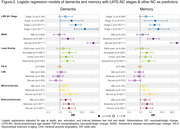# Evaluating updated LATE‐NC staging criteria using National Alzheimer’s Coordinating Center data: stage 1 subtypes matter

**DOI:** 10.1002/alz.086019

**Published:** 2025-01-03

**Authors:** Davis C. Woodworth, Katelynn M. Nguyen, Lorena Sordo, Kiana A. Scambray, Elizabeth Head, Claudia H. Kawas, María M. M. Corrada, Peter T Nelson, S. Ahmad Sajjadi

**Affiliations:** ^1^ University of California, Irvine, Irvine, CA USA; ^2^ The UC Irvine Institute for Memory Impairments and Neurological Disorders (UCI MIND), Irvine, CA USA; ^3^ University of Michigan, Ann Arbor, MI USA; ^4^ College of Medicine, University of Kentucky, Lexington, KY USA

## Abstract

**Background:**

Limbic‐predominant age‐related TDP‐43 encephalopathy neuropathologic change (LATE‐NC) is a common cause of dementia in older age. LATE‐NC was first coined in 2019 with proposed staging criteria of TDP‐43 progressing from amygdala (stage 1), to hippocampus (stage 2), to middle frontal gyrus (stage 3). Criteria were updated in 2023 to further categorize stage 1 to either TDP‐43 inclusions in amygdala alone (stage 1a) or hippocampus alone (stage 1b). We applied this updated LATE‐NC staging criteria to data from participants in the National Alzheimer’s Coordinating Center (NACC) and examined associations with clinical diagnosis and other neuropathologic changes (NCs).

**Method:**

We selected participants in NACC with regional TDP‐43 assessments, excluding those with frontotemporal dementia‐related and rare NCs, or with a non‐Alzheimer’s disease (AD) etiologic clinical diagnosis. LATE‐NC stages were assigned according to updated criteria. We performed logistic regressions with LATE‐NC stages as predictors and clinical diagnosis (dementia, cognitive impairment, memory impairment), other neuropathologic changes (ADNC, Lewy bodies, hippocampal sclerosis of aging (HS‐A), and vascular NCs), or gross atrophy at autopsy (cortical, hippocampal, frontal/temporal lobar) as outcomes. We also examined the association of LATE‐NC stages with dementia and memory impairment while accounting for other NCs.

**Result:**

Of N = 1365 participants, LATE‐NC was present in 519 (37%): 31% stage 1 (78% 1a, 22% 1b), 56% stage 2, 13% stage 3 (Table‐1). Participants with stage 1a were younger at death compared to higher stages. Stage 1a and 1b had similar associations with cognitive problems (Figure‐1). However, while other stages were associated with ADNC and Lewy bodies, stage 1b was not. Meanwhile stage 1b was associated with HS‐A more strongly than stage 1a and to a similar degree as stage 2. We observed expected associations of higher LATE‐NC stages with dementia and other NCs (Figure‐1). Lastly, LATE‐NC stage 1b was significant (and stage 1a trended) for associations with dementia and memory impairment, while higher LATE‐NC stages had associations eclipsed in strength only by highest level ADNC (Figure‐2).

**Conclusion:**

These findings highlight the utility of the updated LATE‐NC staging criteria in general, and stage 1 subtypes in particular, in capturing broad associations with cognitive impairment and specific associations with other neuropathologic changes.